# Changes in foot pain, structure and function following bariatric surgery

**DOI:** 10.1186/s13047-018-0277-y

**Published:** 2018-06-28

**Authors:** Tom P. Walsh, Tiffany K. Gill, Angela M. Evans, Alison Yaxley, Jacob A. Chisholm, Lilian Kow, John B. Arnold, E. Michael Shanahan

**Affiliations:** 1 College of Medicine and Public Health, Flinders University, Bedford Park, South Australia 5042 Australia; 2 Department of Orthopaedics and Trauma, The Queen Elizabeth Hospital, Woodville South, South Australia 5011 Australia; 3 Adelaide Medical School, Faculty of Health and Medical Sciences, The University of Adelaide, Adelaide, South Australia 5005 Australia; 4 Discipline of Podiatry, College of Science, Health and Engineering, La Trobe University, Bundoora, VIC 3086 Australia; 5 Nutrition & Dietetics, College of Nursing and Health Sciences, Flinders University, Bedford Park, South Australia 5042 Australia; 6 Department of Gastrointestinal Surgery, Flinders Medical Centre, Bedford Park, South Australia 5042 Australia; 7 Alliance for Research in Exercise, Nutrition and Activity, Sansom Institute for Health Research, School of Health Sciences, University of South Australia, Adelaide, South Australia 5000 Australia; 8 Department of Rheumatology, Southern Adelaide Local Health Network, Bedford Park, South Australia 5042 Australia

**Keywords:** Foot, Pain, Obesity, Bariatric surgery

## Abstract

**Background:**

Bariatric surgery candidates have a high prevalence of foot pain, depression and elevated plantar pressures. There is, however, limited research into how these factors interact pre- and post-surgery. The aims of this study were therefore to investigate the mechanical and non-mechanical factors associated with foot pain severity before, and the change after, surgery.

**Methods:**

Bariatric surgery candidates underwent baseline and six-month follow-up measures. Foot pain was measured with the Manchester-Oxford Foot Questionnaire. Mechanical measures included body mass index (BMI), dynamic plantar pressures, radiographic foot posture, and hindfoot range of motion. Depressive symptoms, the non-mechanical measure, were assessed by questionnaire. Multivariable linear regression was used to determine which variables were associated with foot pain at baseline and at follow-up. Multilevel repeated models assessed the associations between foot pain and plantar pressure, adjusting for the interaction between group and follow-up time.

**Results:**

Forty-five participants (84% female), with mean (SD) age of 45.7 (9.4) years were recruited. Twenty-nine participants had bariatric surgery and 16 participants remained on the waiting list (controls). Following bariatric surgery, foot pain reduced significantly by - 35.7 points (95% CI -42.2 to - 28.8), while depressive symptoms and whole foot peak pressures had a significant mean change of - 5.9 points (95% CI -10.3 to - 1.5) and - 36 kPa (95% CI -50 to - 22), respectively. In multivariable analysis, depressive symptoms were associated with foot pain at baseline β = 0.7 (95% CI 0.2 to 1.2) after controlling for age, gender, BMI, foot posture and plantar pressure. Depressive symptoms were also associated with foot pain at follow-up in those undergoing bariatric surgery, β = 1.2 (95% CI 0.8 to 1.7). Foot posture and hindfoot range of motion did not change following surgery and a change in plantar pressures was not associated with a change in foot pain.

**Conclusions:**

Foot pain severity in bariatric surgery candidates was associated with depressive symptoms at baseline. Reduced foot pain following bariatric surgery was associated with an improvement in depressive symptoms, without a significant change in foot posture or foot function. Foot pain severity in bariatric candidates may be mediated by non-mechanical or non-local factors before and following surgery.

## Background

Foot pain is a common complaint, affecting almost one in four adults aged over 45 years [1]. Obesity is a risk factor for the development of foot pain [2], and an elevated body mass index (BMI) is strongly associated with both chronic plantar heel pain and non-specific foot pain [3]. Moreover, the feet of people with obesity are structurally and functionally different to their non-obese counterparts, manifesting as thicker, wider and larger, along with flatter-foot postures, reduced joint range of motion and increased peak plantar pressures [4, 5]. It is therefore conceivable that foot pain in people with obesity is related to these mechanical adaptations, particularly the flattening of the foot arches and the increase in plantar pressures.

Studies have found that people with obesity display increases in plantar pressures that are not uniform, with the areas of highest pressure being the midfoot and forefoot, when compared to non-obese people [5–7]. Given that obesity is strongly associated with plantar heel pain [8, 9], increased plantar pressure elsewhere is discordant if pain was strongly related to excessive pressure. Paradoxically, people with chronic plantar heel pain display *reduced* loading under the heel when compared to controls [10]. Indeed, pain may persist even when gait patterns change to offload a painful region of the foot. Thus, chronic foot pain in people with obesity may be more than mechanical overload, involving a complex interplay between mechanical, metabolic and psychological factors.

Musculoskeletal pain has a bidirectional relationship with both obesity [11] and depression [12], while depression and obesity also amplify each other [13]. These relationships, however, are not limited to weight-bearing joints with a known association between elevated BMI and symptomatic hand osteoarthritis [14], suggesting that metabolic mechanisms, including systemic inflammation [15], may underpin the relationship between obesity and joint pain [16]. Whilst these relationships exist in the general population, it is particularly pertinent in bariatric surgery candidates, who are over-represented amongst those complaining of musculoskeletal pain [17]; with foot and ankle pain prevalence cited as 34–50% [18, 19]. There is evidence that spatiotemporal gait patterns, such as increased limb swing and decreased double-limb support time [20] improve following bariatric surgery, but currently only limited investigations regarding associations between weight loss in a bariatric cohort and changes in foot pain, foot function and foot posture. In order to effectively develop and understand treatment methods, it is important to determine whether weight loss has a direct influence on foot structure and function that could be linked with pain, given the high prevalence of obesity across the community [21].

Despite a high prevalence of foot pain, depression and elevated plantar pressures, there is little evidence that mechanical and non-mechanical factors relate to foot pain before and after bariatric surgery. Therefore, the aims of this study were to investigate changes in foot pain, posture and function after bariatric surgery compared to a group remaining on the waiting-list, acting as controls, and to determine the factors related to changes in foot pain post-surgery.

## Methods

### Participants

This study recruited a convenience sample of people with foot pain from bariatric surgery waiting lists at two tertiary hospitals in Adelaide, South Australia between January 2015 and June 2017. All patients on the lists were invited to participate in this study. Participants were recruited either immediately before surgery (treatment group) or when they were added to the waiting list (control group). All participants underwent baseline measures and were reassessed at six-month follow-up. Baseline measures for participants in the treatment group were recorded 2–3 weeks prior to surgery, designed to reduce the impact of non-surgical weight loss that occurs with meal replacements prior to surgery. Bariatric surgery procedures (sleeve gastrectomy, laparoscopic adjustable gastric band or Roux-en-Y gastric bypass) were based on the clinical requirements of each patient, and not directed by this study. The study received ethical approval before commencement by the Southern Adelaide Clinical Human Research Ethics Committee (Project ID 211.14).

### Inclusion and exclusion criteria

People aged ≥18 years were recruited if they reported current foot pain of ≥30 mm on a visual analogue scale [22], indicating moderate pain (at least), which had to have been present for ≥3 months. Participants were excluded if: pregnant, history of previous bariatric or foot surgery, systemic inflammatory disease, loss of peripheral sensation in the feet, known infectious disease, cancer, non-ambulatory.

### Demographic and anthropometric data

Self-reported age and gender were recorded at baseline. Body weight and height were measured to the nearest 0.1 kg and 0.1 cm, respectively using an electronic stadiometer (with shoes, socks, and bulky clothing removed) (Seca 284, Germany). From these data, BMI (weight (kg)/height (m^2^)) was calculated [23].

### Foot pain

Foot pain and disability were assessed using the Manchester-Oxford Foot and Ankle Questionnaire (MOXFQ). The MOXFQ is a reliable and valid 16-item questionnaire that comprises three separate underlying dimensions: walking/standing problems (seven items), foot pain (five items), and social interaction (four items). Item responses are each scored from 0 to 4, with 4 representing the most severe state [24]. The MOXFQ-index is a summary score that incorporates all three domains and was used for this study as it investigates overall foot pain and disability and is graded on a 0 to 100 scale, with 100 being the most severe state.

### Depressive symptoms

The Center for Epidemiologic Studies Depression scale (CES-D) consists of 20-items that assess depressive symptoms [25]. All items are graded via a four-point (0 to 3) Likert scale, giving a possible range of 0–60. The sum score was used as a continuous variable to assess depressive symptoms at baseline and follow-up.

### Plantar pressure

Dynamic plantar pressure data were collected with the MatScan® (Tekscan, USA) platform system at baseline and follow-up. The platform is a 5 mm-thick floor mat (432 × 368 mm) incorporating 2288 resistive sensors (sensor size = 0.70 cm^2^, 1.4 sensors/cm^2^) with data sampled at a rate of 40 Hz. Analyses conducted using the MatScan® platform have been previously shown to have good accuracy [26] and moderate to good inter-session reliability in adults, for total peak pressure and maximum force ICC (95% CI) of 0.58 (0.28 to 0.75) and 0.92 (0.84 to 0.96), respectively [27]. The MatScan® platform was positioned in a level walkway. Following individual step calibration, which involved entering the participant’s body weight followed by a period of standing on the MatScan® platform, balancing on one foot. The participants were then asked to walk barefoot with their usual gait pattern across the MatScan® platform. Data were collected using a midgait protocol, whereby participants were instructed to take two steps, striking the platform on their third step, before continuing to walk for a further three steps. The midgait protocol has been found to have moderate to excellent reliability, for total peak pressure and maximum force ICC of 0.52 to 0.97 and 0.36 to 0.73, respectively [28]. Data were collected from three complete, valid trials of the right foot. A valid trial was determined when participants’ gait pattern was not perturbed and when no ‘targeting’ of the plantar pressure platform occurred. Individual “masks” were manually constructed to determine plantar pressures for the whole foot and also five regions of the foot; heel, midfoot, forefoot, hallux and lesser toes, using the Research Foot software (version 6.51). Measures of regional contact area (cm^2^), maximum force (kgf), peak plantar pressure (kPa) and contact time (ms) were calculated for all trials to obtain a mean value. The right foot was chosen to ensure that the assumption of independence of data was met [29] and additional measures such as mean pressure or pressure-time integral were not collected given the interdependence between these measures and peak pressure [30, 31]. Contact time was used as a proxy for walking speed [32].

### Radiographic measures

Weight-bearing dorsoplantar and lateral radiographs were taken at baseline and follow-up using a standardised technique on a digital radiograph unit (Ysio, Siemens Healthcare, Germany). Dorsoplantar views were taken with the participant standing above the image receptor on both feet, the central beam was angled at 15 degrees towards the calcaneus and the centering point was aimed at the base of the third metatarsal. Lateral radiographs were taken with the participant standing on a platform, the image receptor was positioned on the medial aspect of the foot and the centering point was at the base of the metatarsals from a lateral to medial direction.

Foot posture was assessed using four radiographic angles described by Murley et al. [33]. The calcaneal inclination angle is the angle between the inferior aspect of the calcaneus and the supporting surface, while the calcaneal-first metatarsal angle is the angle on the dorsum of the foot taken between the inferior calcaneal angle and a line parallel to the midshaft of the first metatarsal. Both of these angles were measured from the lateral view. The calcaneal-first metatarsal angle was used as a measure of foot posture in the regression analyses, having been shown to strongly correlate with clinical measures [33].

Two further angles were taken from the dorso-plantar radiograph: the talo-navicular and talo-second metatarsal angle. The anteromedial and anterolateral extremes of the talar head and the bisection of the proximal articular surface of the navicular formed the talo-navicular angle. The talo-second metatarsal angle is formed between the bisection of the shaft of the second metatarsal and the line perpendicular to the anteromedial and anterolateral extremes of the head of the talus.

### Hindfoot range of motion

Ankle joint dorsiflexion was measured using the technique described by Munteanu et al. [34], which has very good intra- and inter-rater reliability (ICC (95% CI) 0.88 (0.75–0.94) and 0.92 (0.86–0.96)) when taken in a weight-bearing position with the knee in full extension. A digital protractor (Gain Express, USA) was placed on the anterior aspect of the tibia and the angle between the ground and the leg was recorded. The measure was repeated in duplicate and a mean was obtained.

Frontal plane range of motion of the hindfoot (ankle and subtalar joints) was measured using the technique described by Menadue et al. [35]. Participants are seated in an upright position with the foot positioned overhanging the examination table. The foot was moved from maximal abduction to maximal adduction with the total range of motion recorded with a goniometer (Physio-Med, AUS), this measure was repeated in duplicate and a mean was obtained.

### Data analysis

Descriptive statistics (frequencies and means) were obtained. All data distributions were checked for normality via the inspection of histograms and the Shapiro-Wilks test prior to inferential statistical analysis. Differences between the treatment and control groups at baseline were assessed using chi-squared tests for categorical data and t-tests or Mann-Whitney *U* tests for continuous data that was normally or non-normally distributed, respectively. Multivariable linear regression was used to analyse the association between foot pain severity and mechanical (foot posture, plantar pressure, BMI) and non-mechanical (depressive symptoms) variables, along with age and gender at baseline and follow-up. Within group differences in contact area, force and peak plantar pressure between baseline and follow-up were analysed with either the paired-samples t-test or the Wilcoxon signed-rank test. As repeated measures were taken over six-months, a multilevel repeated model using the xtmixed command in STATA was used to assess the associations between MOXFQ-index and peak plantar pressure and other covariates (ankle joint range of motion [36], contact time [37], age and gender [38]) for both groups over six-months while adjusting for the interaction between group and follow-up time. This type of regression model allows for the correlations of observations within subjects. In all analyses, *p* values (two-sided) less than 0.05 were deemed to be statistically significant. All data analyses were performed with SPSS V25 (IBM SPSS Statistics, Armonk, NY, USA) and STATA V15 (StataCorp College Station, TX, USA).

## Results

### Participant characteristics

This study recruited 45 participants (38 women), with a mean (standard deviation) (SD) age of 45.7 (9.4) years. Twenty-nine participants had bariatric surgery, undergoing a Roux-en-Y gastric bypass (*n* = 13), sleeve gastrectomy (*n* = 11), or laparoscopic adjustable gastric band (*n* = 5). Sixteen participants remained on the waiting-list and were used as controls. There were no significant differences in the characteristics between those undergoing bariatric surgery and those in the control group (Table 1). All participants underwent baseline measures, one participant who underwent bariatric surgery was lost to follow-up as they were uncontactable. Two additional participants from the treatment group were unavailable for follow-up plantar pressure data collection and four participants from the control group were unavailable for follow-up plantar pressure and foot posture data collection.

**Table 1:** Baseline characteristics of the treatment and control groups^^a^

	Treatment group (*n* = 27)	Control group (*n* = 16)	*p* value
Age, years	45.1 (9.0)	45.3 (10.4)	0.958
Gender, women, *n* (%)^b^	25 (86.2)	13 (81.3)	0.661
Height, m	1.7 (0.1)	1.7 (0.1)	0.906
Weight, kg	123.9 (19.4)	132.4 (15.5)	0.140
Body mass index, kg/m^2^	44.8 (7.0)	47.9 (5.2)	0.120
MOXFQ-index, points	49.7 (18.5)	61.0 (23.0)	0.081
Sum CES-D score	16.9 (11.6)	22.3 (12.0)	0.151
Plantar pressures			
Contact area (cm^2^)			
Whole foot	181.2 (26.7)	185.2 (23.2)	0.625
Forefoot^c^	62.3 (9.3)	63.5 (8.6)	0.910
Hallux^c^	34.0 (6.9)	35.9 (13.3)	0.940
Heel	54.0 (8.6)	55.1 (5.0)	0.659
Lesser toes	31.8 (5.6)	31.7 (6.9)	0.952
Midfoot	45.8 (10.6)	46.9 (6.1)	0.658
Force (kgf)			
Whole foot	149.5 (31.5)	155.7 (17.5)	0.479
Forefoot	97.3 (20.6)	99.8 (14.4)	0.669
Hallux^c^	33.9 (7.2)	36.1 (13.1)	0.950
Heel	80.7 (19.6)	85.8 (10.8)	0.284
Lesser toes	31.2 (7.5)	30.8 (9.1)	0.870
Midfoot	41.4 (11.3)	41.6 (8.9)	0.953
Plantar pressure (kPa)			
Whole foot^c^	386 (50)	393 (34)	0.920
Forefoot	366 (56)	370 (41)	0.785
Hallux	170 (43)	186 (74)	0.437
Heel	354 (61)	365 (56)	0.561
Lesser toes	54 (20)	60 (22)	0.375
Midfoot	181 (77)	197 (55)	0.472
Contact time (seconds)	0.9 (0.1)	0.9 (0.1)	0.745
Hindfoot ROM (degrees)			
Frontal plane^c^	40.9 (6.3)	38.3 (4.7)	0.094
Dorsiflexion	61.2 (5.0)	62.4 (6.4)	0.492
Foot posture (degrees)			
Talo-second metatarsal angle	12.0 (8.4)	8.8 (10.1)	0.265
Talo-navicular angle	15.3 (6.9)	11.4 (7.6)	0.092
Calcaneal inclination angle^c^	20.6 (4.9)	22.1 (3.6)	0.445
Calcaneal-first metatarsal angle	133.1 (6.3)	131.6 (6.0)	0.420

*SD* standard deviation*, m* metres*, kg* kilograms*, m*^*2*^ metres squared, *cm*^*2*^ centimetres squared*, CES-D* Center for Epidemiological Studies-Depression scale, *MOXFQ* Manchester-Oxford Foot Questionnaire, *kPa* kilopascal, *ROM* range of motion

^a^*p* calculated for differences between the treatment and control groups analysed with the independent samples t-test

^b^*p* calculated for differences between the treatment and control groups analysed with the chi-squared test

^c^*p* calculated for differences between the treatment and control groups analysed with the Mann-Whitney *U* test

^^^values are mean (SD) unless otherwise stated

### Baseline and follow-up foot pain

In multivariable regression analysis, only depressive symptoms were significantly associated with foot pain severity measured by MOXFQ at baseline (β = 0.7, 95% CI 0.2 to 1.2, *p* = 0.008). Age, gender, BMI, foot posture and whole-foot plantar pressure were included in the model, but these were not significantly related to foot pain severity, although age and BMI were trending towards statistical significance (Table 2). Multivariable regression analysis of the treatment group at follow-up found foot pain severity was also associated with depressive symptoms (β = 1.2, 95% CI 0.8 to 1.7, *p* <  0.001), while age, gender, foot posture and whole foot plantar pressure were not significantly associated with foot pain. There were no significant associations between foot pain severity and any of the listed variables in the control group (Table 3). The treatment group had a significant reduction in foot pain of 35.7 points (95% CI -42.2 to − 28.8, *p* <  0.001), while the control group had a small non-significant reduction of 4.0 points (95% CI -15.2 to 7.1, *p* = 0.454) (Table 4). Nine people (one-third) in the treatment group had complete resolution of their foot pain at 6-months follow-up.

**Table 2:** Multivariable relationships with MOXFQ-index at baseline for all participants (*n* = 43)

	β-coefficients (95% CI)	*p* value
Age	0.6 (− 0.1 to 1.4)	0.095
Gender (female)	− 0.5 (− 17.2 to 16.3)	0.954
Body mass index	1.0 (− 0.0 to 2.0)	0.052
Depressive symptoms^a^	0.7 (0.2 to 1.2)	0.008
Foot posture^b^	−0.6 (− 1.5 to 0.3)	0.206
Plantar pressure^c^	−0.1 (− 0.2 to 0.1)	0.424

*MOXFQ* Manchester-Oxford Foot Questionnaire, *CI* confidence interval

^a^ sum Center for Epidemiological Studies-Depression scale score

^b^ calcaneal-first metatarsal angle

^c^ whole foot peak pressure

**Table 3:** Multivariable relationships with MOXFQ-index at follow-up for the treatment group and the control group

	Treatment group (*n* = 26)		Control groups (*n* = 12)	
	β-coefficients (95% CI)	*p* value	β-coefficients (95% CI)	*p* value
Age	0.2 (− 0.3 to 0.8)	0.434	0.7 (− 2.2 to 3.6)	0.582
Gender (female)	−1.1 (− 14.3 to 12.0)	0.858	19.1 (− 56.5 to 94.6)	0.545
Body mass index	0.4 (−0.3 to 1.1)	0.280	1.3 (− 4.8 to 7.4)	0.609
Depressive symptoms^a^	1.2 (0.8 to 1.7)	< 0.001	1.3 (−1.1 to 3.7)	0.229
Foot posture^b^	−0.2 (− 0.8 to 0.4)	0.515	0.3 (− 5.9 to 6.5)	0.915
Plantar pressure^c^	0.1 (−0.0 to 0.1)	0.291	−0.0 (− 1.2 to 1.2)	0.959

*MOXFQ* Manchester-Oxford Foot Questionnaire, *CI* confidence interval

^a^sum Center for Epidemiological Studies-Depression scale score

^b^calcaneal-first metatarsal angle

^c^whole foot peak pressure

**Table 4:** Comparison between baseline and six-month follow-up measures of body weight, foot pain, depressive symptoms, plantar pressure, foot posture and ankle joint range of motion for treatment and control groups^a^

	Treatment group (*n* = 26)			Control group (n = 12)		
	Mean difference	95% CI	*p* value	Mean difference	95% CI	*p* value
Weight, kg	− 24.3	− 27.5 to − 21.1	< 0.001	1.2	−2.5 to 4.9	0.877^b^
Body mass index, kg/m^2^	− 8.8	− 10.0 to − 7.6	< 0.001	0.5	−0.8 to 1.8	0.440
MOXFQ-index, points	−35.7	−42.7 to − 28.8	< 0.001	− 4.0	− 15.2 to 7.1	0.454
Sum CES-D score	−5.9	− 10.3 to − 1.5	0.007^b^	− 4.6	−9.6 to 0.3	0.061^b^
Contact area (cm^2^)						
Whole foot	−3.4	−8.7 to 2.0	0.210	−3.8	−6.7 to − 0.9	0.014
Forefoot	−0.1	−2.1 to 1.9	0.427^b^	0.7	−0.6 to 2.1	0.239^b^
Hallux	1.0	−0.7 to 2.8	0.232	1.8	−3.3 to 6.9	0.844^b^
Heel	− 1.4	− 3.4 to 0.6	0.155	− 0.4	−2.0 to 1.2	0.579
Lesser toes	−0.0	−1.8 to 1.7	0.960	1.2	−1.3 to 3.8	0.306
Midfoot	−2.1	−3.9 to −0.3	0.022	0.0	−1.5 to 1.6	0.946
Force (kgf)						
Whole foot	−30.2	−36.3 to −24.2	< 0.001	7.1	−2.4 to 16.6	0.126
Forefoot	−18.7	−24.4 to − 13.0	< 0.001^b^	6.0	0.7 to 11.3	0.010^b^
Hallux	1.5	−0.9 to 3.9	0.215	−0.3	−2.7 to 2.1	0.724^b^
Heel	− 13.9	−18.2 to − 9.5	< 0.001	1.5	− 3.0 to 6.1	0.477
Lesser toes	−0.0	−1.8 to 1.7	0.961	0.6	−1.6 to 2.7	0.581
Midfoot	−6.5	−9.2 to −3.9	< 0.001	0.1	−3.3 to 3.5	0.930
Plantar pressure (kPa)						
Whole foot	−36	−50 to − 22	< 0.001	13	0 to 25	0.046
Forefoot	−40	−56 to − 25	< 0.001	123	−3 to 28	0.098
Hallux	−16	−31 to − 2	0.031	3	−15 to 21	0.705
Heel	−45	−65 to − 25	< 0.001	10	−10 to 30	0.276
Lesser toes	−3	−9 to 3	0.366	3	−9 to 16	0.456^b^
Midfoot	− 45	− 63 to − 26	< 0.001	3	− 14 to 20	0.709
Contact time (seconds)	− 0.0	−0.0 to 0.0	0.617	0.0	−0.0 to 0.0	0.553
Hindfoot ROM (degrees)						
Frontal plane	−1.4	−4.2 to 1.4	0.410^b^	1.8	−0.3 to 3.8	0.090^b^
Dorsiflexion	0.5	−2.4 to 3.4	0.748	0.5	−2.0 to 3.0	0.643
Foot posture (degrees)						
Talus-second metatarsal angle	−0.2	−1.8 to 1.5	0.441^b^	1.3	−0.1 to 2.6	0.063
Talo-navicular angle	−1.4	−3.1 to 0.3	0.095^b^	1.0	−1.0 to 3.0	0.284
Calcaneal inclination angle	0.1	−0.3 to 0.6	0.623^b^	0.1	−0.7 to 0.9	0.665^b^
Calcaneal-first metatarsal angle	−0.4	− 0.3 to 1.0	0.300	− 0.4	− 1.9 to 1.0	0.539

*m* metres*, kg* kilograms*, m*^*2*^ metres squared, *cm*^*2*^ centimetres squared*, MOXFQ* Manchester-Oxford Foot Questionnaire, *kPa* kilopascal, *ROM* range of motion, *CES-D* Center for Epidemiological Studies-Depression scale

^a^*p* calculated for differences between baseline and follow-up measures analysed with the paired samples t-test unless otherwise stated

^b^*p* calculated for differences between baseline and follow-up measures analysed with the Wilcoxon signed-rank test

### Change in mechanical and non-mechanical variables following bariatric surgery

**Fig. 1:**
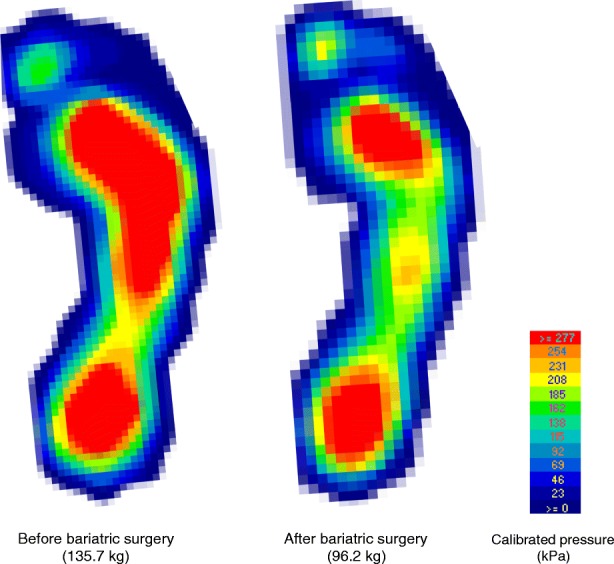
Representative peak plantar pressures during walking before and 6-months following bariatric surgery

The 29 participants who underwent bariatric surgery had a mean reduction in weight of 24.3 kg (95% CI -27.5 to − 21.1, *p* <  0.001), representing a mean per cent change in body weight of 19.9% (range 0.9 to 31.8%). The 16 participants who did not have bariatric surgery had a mean weight gain of 1.2 kg (95% CI -2.5 to 4.9, *p* = 0.877). In the treatment group, there were significant reductions in force and peak pressure in multiple regions, although the midfoot was the only site to exhibit a statistically significant mean reduction in contact area of 2.1 cm^2^ (95% CI -3.9 to − 0.3, *p* = 0.022). Representative peak plantar pressures of participants before and after bariatric surgery are depicted in Fig. 1. In the control group, the only region to demonstrate a statistically significant change in plantar pressure was the whole foot variable, which increased by a mean 13 kPa (95% CI 0 to 25, *p* = 0.046). Depressive symptoms reduced in both groups, although only the treatment group had a significant mean reduction of 5.9 points (95% CI -10.3 to − 1.5, *p* = 0.007). There was no significant change in the ankle joint range of motion or foot posture in either the treatment group or the control group between baseline and follow-up. The crude changes between baseline and follow-up are detailed in Table 4.

### Repeated measures analysis for change in MOXFQ-index and plantar pressure

The change in foot pain severity between baseline and follow-up was not associated with peak pressure when adjusted for other covariates. Both group (β = − 11.2, 95% CI -23.8 to 1.4, *p* = 0.081) and follow-up time (β = − 7.7, 95% CI -18.5 to 3.2, *p* = 0.168) variables were associated with a reduction in foot pain, but only the group*time (6-months) interaction was statistically significant (β = − 21.1, 95% CI -40.8 to − 13.5, *p* <  0.001) (Table 5).

**Table 5:** Repeated measures analysis of change in MOXFQ-index and plantar pressure

Variable	β-coefficients (95% CI)	*p* value
Age	0.5 (−0.1 to 1.2)	0.123
Gender	12.0 (−1.3 to 25.4)	0.077
Peak pressure^a^	0.0 (− 0.1 to 0.1)	0.615
Ankle joint dorsiflexion	0.3 (−0.7 to 1.3)	0.541
Contact time	18.4 (−17.9 to 54.7)	0.320
Group (treatment)	−11.2 (− 23.8 to 1.4)	0.081
Time (6-months)	−7.7 (−18.5 to 3.2)	0.168
Group*time (6-months)	−27.1 (−40.8 to − 13.5)	< 0.001

^a^Whole foot peak pressure

## Discussion

This is the first study to comprehensively examine the effect of weight loss following bariatric surgery on foot pain, and to explore the mechanical and non-mechanical factors associated with foot pain severity. Depressive symptoms were associated with foot pain severity at baseline, after accounting for age, gender, BMI, foot posture and plantar pressure. At follow-up, foot pain severity was associated with depressive symptoms in those who had undergone bariatric surgery. The change in plantar pressure, walking speed or ankle joint dorsiflexion was not associated with a change in foot pain following bariatric surgery, but weight loss following bariatric surgery resulted in a significant reduction in foot pain severity at 6-months. Therefore, in this cohort, both baseline foot pain and change in foot pain appear more strongly related to non-mechanical or non-local factors.

Previous studies examining the association between the change in weight and change in plantar pressure have largely focused on the effect of weight gain. The effect of weight gain on plantar pressures has been frequently performed on asymptomatic participants, using weighted backpacks as a proxy for the increase in weight [8, 39]. This method, while practical, measures the instantaneous effect of a change in weight and does so in asymptomatic feet, and therefore may not accurately reflect how pressures change over time in symptomatic feet. This method is also impractical in assessing weight loss. Investigations analysing the effects of weight loss are limited, although a randomised controlled trial investigated the effects of non-surgical weight loss on plantar pressures, albeit in asymptomatic participants, and found that even a small amount of weight loss can significantly reduce mean peak plantar pressure across multiple regions of the foot [40]. This study found that larger weight loss following bariatric surgery results in widespread reductions in plantar pressures in symptomatic feet, with the largest reduction in plantar pressure found in the midfoot.

Interestingly, the reduction in midfoot pressure in the treatment group occurred without a significant change in radiographic foot posture and may therefore be related to soft tissue changes. A previous cross-sectional study found that people with obesity have increased three-dimensional foot circumference at multiple sites when compared to people with a healthy weight [41], these differences are likely soft tissue related. Moreover, the change in BMI and midfoot pressure is concordant with a previous study investigating weight gain and plantar pressure [42], and suggests that the midfoot may be a region that is the most responsive to a change in weight. The reduction of force in the midfoot in our study may have resulted in a larger reduction in peak pressure, if it were not for the significant simultaneous reduction in contact area of the midfoot. Furthermore, a study investigating contact area and body composition found a positive association between total body fat mass, but not fat-free mass, and the midfoot contact area only [43]. Together, these findings suggest that people with higher fat mass may deposit fat mass in the midfoot and, following bariatric surgery, there may be a loss of this fat mass that could appear to elevate the longitudinal arch of the foot. This may have implications for the fit of footwear or orthoses following soft tissue adaptations after bariatric surgery, and in patients with significant weight loss.

The association between foot pain severity and depressive symptoms has previously been established in a community cohort [44], suggesting that foot pain may be a manifestation of either widespread or reduced threshold for pain that extends beyond localised discomfort. The results of our study are concordant with this premise, although ours are unique given they are exclusively from a bariatric cohort and we were able to adjust for local foot measures, including foot posture and plantar pressure at baseline and follow-up. Given the high prevalence of depression in bariatric surgery candidates, and the improvement in depressive symptoms following surgery [45], it is possible that foot pain severity is mediated by depressive symptoms, rather than by body weight alone. There is evidence that while depressive symptoms improve in the short-term following bariatric surgery, there may be attenuation of this improvement in the longer term, and indeed some people have increased depressive symptoms following surgery, often with concomitant weight regain [46]. Whether this causes an exacerbation of musculoskeletal pain is not known, but this may be worth exploring.

This study should be considered in light of some limitations. Firstly, the small sample size limited the number of variables we could include in our models and may have may resulted in type II errors for the variables we did include. Secondly, the cohort was recruited via convenience sampling and consisted of mainly women which may limit the generalisability (and the ability to analyse between gender comparisons) of the findings, however, this is consistent with the demographics that present for bariatric surgery thus findings are applicable to that context [47]. Thirdly, the spatial resolution of the MatScan® plantar pressure system is relatively low and thus the sensitivity to detect all changes in plantar pressure may have been compromised. Furthermore, the importance of the size of the sensors used in plantar pressure systems has also been well described [48], and this may have impacted on measuring contact area, particularly for the lesser toe region, which is prone to measurement error [49]. Whilst there are limitations regarding sensor size and spatial resolution, the detection of subtle changes in plantar pressure was less important given the gross changes in body mass (and pressure) that occurs following bariatric surgery. Fourthly, the duration of foot pain was not recorded, so there may be variation of pain duration prior to participant enrolment. Finally, the change in foot pain and plantar pressure was measured over a six-months, and may not reflect changes seen over longer periods.

Nonetheless, this study has a number of strengths. It is the first to examine foot pain, foot posture and plantar pressures in bariatric candidates, reporting the effect of bariatric surgery on all variables. This study also considered relationships between mechanical and non-mechanical factors and foot pain severity at baseline and prospectively following bariatric surgery.

The results of this study provide proof of concept that weight loss improves foot pain, and future studies in this area, including non-surgical weight loss strategies and less obese cohorts, may be warranted. Deeper analysis of gait characteristics before and following weight loss in people with foot pain, may also determine if changes occurring beyond peak plantar pressures are important to consider in this cohort, and even footwear choices may be relevant.

## Conclusion

Foot pain significantly improves following bariatric surgery instigated weight loss, but occurs without a change in foot structure. Dynamic peak plantar pressures reduce following bariatric surgery and weight loss, but these changes are not related to changes in pain. Depressive symptoms, however, are significantly related to foot pain both before and following bariatric surgery and associated weight loss. Thus, foot pain in bariatric candidates may be mediated by non-mechanical or non-local factors before and following surgery and resulting weight loss.

## Abbreviations

AUS: Australia
BMI: body mass index
CES-D: Center for Epidemiologic Studies Depression
CI: confidence interval
cm: centimetre
HREC: Human Research Ethics Committee
Hz: Hertz
IBM: International Business Machines Corporation
ICC: Intraclass correlation coefficient
kg: kilogram
kPa: kilopascal
m: metre
MOXFQ: Manchester-Oxford Foot Questionnaire
ms: millisecond
NY: New York
SD: standard deviation
SPSS: Statistical Package for the Social Sciences
TX: Texas
USA: United States of America
